# Eltrombopag for Chemotherapy-Induced Thrombocytopenia: A Multicenter Retrospective Real-World Study

**DOI:** 10.3390/hematolrep18030039

**Published:** 2026-06-10

**Authors:** Mehmet Baysal, Fatos Dilan Köseoğlu, Sevil Sadri, Ünal Ataş, Ufuk Demirci, Rafiye Çiftçiler, Seval Akpınar, Elif Gülsüm Ümit

**Affiliations:** 1 Division of Hematology, Department of Internal Medicine, Tekirdag Namık Kemal University, 59030 Tekirdag, Türkiye; 2 Division of Hematology, Department of Internal Medicine, İzmir Bakircay University, 35665 İzmir, Türkiye; 3 Hematology Clinic, Bursa City Hospital, 16110 Bursa, Türkiye; 4 Division of Hematology, Department of Internal Medicine, Akdeniz University, 07070 Antalya, Türkiye; vrlunalatas@gmail.com; 5 Division of Hematology, Department of Internal Medicine, Manisa Celal Bayar University, 45140 Manisa, Türkiye; 6 Division of Hematology, Department of Internal Medicine, Selçuk University, 42131 Konya, Türkiye; 7 Division of Hematology, Department of Internal Medicine, Trakya University, 22030 Edirne, Türkiye

**Keywords:** chemotherapy-induced thrombocytopenia, thrombopoietin receptor agonist, eltrombopag, supportive care, dose intensity

## Abstract

**Introduction:** Chemotherapy-induced thrombocytopenia (CIT) is a common dose-limiting toxicity that disrupts on-time, full-dose chemotherapy, yet no pharmacologic therapy is formally approved. Growing evidence from randomized and late-phase studies with thrombopoietin receptor agonists (TPO-RAs) has renewed interest in targeted supportive care. We evaluated the effectiveness and safety of eltrombopag for CIT in routine clinical practice. **Methods**: We conducted a small, retrospective, single-arm multicenter cohort study of 31 adults with solid tumors (74.2% stage IV). Given the descriptive, hypothesis-generating nature of this study, no causal inference regarding efficacy can be drawn. Platelet counts and chemotherapy continuity were tracked from baseline through week 12 after eltrombopag initiation. Bleeding, thrombosis, and laboratory safety signals were recorded. **Results**: The median platelet count increased from 33 × 10^9^/L at baseline to 71 × 10^9^/L at week 1 and 99.5 × 10^9^/L by week 12. Overall, 18/31 patients (58.1%) resumed chemotherapy within 3 weeks, and 15/31 (48.4%) completed planned regimens by week 6. Adverse events were limited to mild, transient elevations in transaminases (*n* = 3); no major bleeding or thrombotic events occurred. **Conclusions**: In this real-world multicenter cohort, eltrombopag was associated with rapid platelet recovery and improved chemotherapy deliverability with an acceptable safety profile. The retrospective, single-arm design and the hypothesis-generating nature of these findings preclude definitive conclusions regarding causal efficacy. These observational data highlight the need for prospective controlled trials to characterize the clinical role, optimal dosing, and long-term safety of oral TPO-RAs in CIT.

## 1. Introduction

Chemotherapy-induced thrombocytopenia (CIT) is a common toxicity that may limit the delivery and intensity of cytotoxic chemotherapy, for which no pharmacologic therapy is formally approved. Management remains largely supportive with transfusion and chemotherapy modification. In routine oncology practice, chemotherapy is typically delayed or dose-reduced when platelet counts decline toward 75–100 × 10^9^/L, a threshold reflected in many protocols despite limited evidence supporting its necessity [1,2]. Such CIT-driven treatment modifications reduce relative dose intensity (RDI), a parameter consistently associated with survival across solid tumors. Multiple large studies and meta-analyses have demonstrated that even modest decreases in RDI negatively affect outcomes in breast, lung, colorectal, and gynecologic cancers, underscoring that a CIT episode is not a benign occurrence but a potential turning point in the systemic treatment trajectory [3,4,5,6,7,8]. CIT therefore remains the only common chemotherapy-related cytopenia without an accepted, guideline-level supportive treatment.

Given the absence of an approved supportive treatment, thrombopoietin receptor agonists (TPO-RAs) have emerged as a biologically plausible strategy to restore megakaryopoiesis, raise platelet counts, and preserve chemotherapy intensity. Contemporary guidelines from the ISTH Subcommittee on Hemostasis and Malignancy acknowledge that carefully selected CIT patients may receive off-label TPO-RA therapy, provided that individualized risk–benefit assessment and thrombotic monitoring are ensured [2,9,10]. In addition, treatment patterns for TPO-RAs may vary across countries according to regulatory approval, reimbursement, drug availability, and physician preference. A recent Delphi-based national consensus study from Türkiye in adult primary immune thrombocytopenia likewise highlighted variability in the practical use, sequencing, and dose optimization of TPO-RAs in routine hematology practice, underscoring that real-world use is shaped not only by evidence but also by local practice conditions and access considerations [11].

Among TPO-RAs, the strongest clinical evidence comes from romiplostim. Randomized and real-world multicenter studies have shown that romiplostim corrects CIT in approximately 70–80% of patients, reduces chemotherapy delays and dose reductions, and does not increase thromboembolic risk [12,13,14,15]. Complementing these data, a large multicenter, real-world cross-sectional study from China evaluated several thrombopoietic agents, including recombinant human TPO and oral TPO-Ras, and likewise reported improved platelet recovery without identifying new safety concerns [16]. Together, these findings form the rationale underpinning recent reviews and consensus statements on selective TPO-RA use in CIT [17,18].

Evidence for eltrombopag in CIT is comparatively more limited but biologically consistent. Phase 1 and phase 2 randomized studies in patients receiving gemcitabine- or carboplatin/paclitaxel-based chemotherapy have demonstrated that eltrombopag increases pre-chemotherapy platelet counts, reduces the incidence of grade 3–4 thrombocytopenia, and decreases chemotherapy delays—although, similar to avatrombopag, some composite primary endpoints were not met due to substantial spontaneous recovery in placebo arms [19,20,21]. Narrative reviews and expert commentaries have emphasized that TPO-RA benefit is most likely in patients with persistent or recurrent CIT, prior pelvic irradiation, marrow infiltration, or exposure to inherently thrombocytopenic regimens such as platinum and gemcitabine [17,22,23].

In Türkiye, eltrombopag is approved for immune thrombocytopenia and severe aplastic anemia, whereas romiplostim is approved only for immune thrombocytopenia. This regulatory landscape has led to selective off-label use of eltrombopag in challenging CIT scenarios—particularly in patients for whom chemotherapy delays are clinically unfavorable and spontaneous recovery is unlikely. However, real-world data on eltrombopag use in heterogeneous, heavily pretreated solid tumor populations, particularly regarding dosing strategies and chemotherapy deliverability remain limited. In this retrospective multicenter study, we evaluated eltrombopag for CIT in routine oncology practice, focusing on platelet recovery, chemotherapy deliverability, and safety.

## 2. Methods

### 2.1. Study Design and Setting

We conducted a retrospective, multicenter cohort study across seven oncology/hematology centers in Türkiye, including consecutive adult patients treated between 1 January 2021 and 31 May 2024. The data cut-off was May 2024. The study was approved by the Trakya University Faculty of Medicine Non-Interventional Research Ethics Committee (TUTF-GOBAEK 2024/48) and conducted in accordance with the Declaration of Helsinki.

### 2.2. Patients

A total of 31 consecutive patients who met the inclusion criteria were enrolled in the study. Eligible patients were ≥18 years old, had a histologically confirmed solid tumor receiving systemic chemotherapy, and developed chemotherapy-induced thrombocytopenia (CIT) for which eltrombopag was prescribed. CIT was defined as a platelet count <75 × 10^9^/L measured at the start of a planned chemotherapy cycle, typically 3–4 weeks after the previous cycle, in the absence of alternative causes of thrombocytopenia (e.g., marrow infiltration, sepsis, disseminated intravascular coagulation, recent major bleeding). All patients had persistent thrombocytopenia, placing them at risk for chemotherapy delay or dose reduction. Persistent thrombocytopenia was defined as failure of platelet count to recover to ≥75 × 10^9^/L by the time of the next planned chemotherapy cycle (typically 3–4 weeks after the previous cycle). Most had advanced disease (stage IV) and prior systemic therapy and/or radiotherapy. Bone marrow biopsy was not systematically performed across participating centers, which represents an important limitation of this retrospective analysis. Evaluation of marrow involvement relied on clinical assessment and radiological imaging, primarily PET/CT. We acknowledge that PET/CT is not equivalent to bone marrow biopsy, as certain solid tumor infiltrations may be PET-negative and diffuse marrow uptake patterns can be nonspecific.

### 2.3. Data Collection

Demographic characteristics, tumor type, systemic therapy details, prior radiotherapy or alkylator exposure, baseline laboratory values, eltrombopag dose and duration, and concomitant medications were extracted from electronic medical records using a standardized data collection form.

### 2.4. Eltrombopag Exposure

Eltrombopag was used off-label for CIT following authorization by the Turkish Medicines and Medical Devices Agency. Treatment was initiated at 50 mg once daily, typically 5–7 days before the planned chemotherapy cycle, at the discretion of the treating physician. Because all patients had established CIT, eltrombopag use was considered therapeutic rather than prophylactic. Dose adjustments were permitted to maintain platelet counts within target ranges or to address laboratory abnormalities.

### 2.5. Outcomes

Primary outcomes were: (i) achievement of a platelet count ≥75 × 10^9^/L at predefined intervals (week 1, week 3–4, week 6, week 12), and (ii) ability to resume or continue planned chemotherapy without delay. A platelet threshold of ≥75 × 10^9^/L was used to define response, reflecting a clinically meaningful level at which chemotherapy may be continued or resumed in routine practice, even if not always at full dose. This threshold is also consistent with the Common Terminology Criteria for Adverse Events (CTCAE), in which platelet counts between 75 and <100 × 10^9^/L correspond to grade 1 thrombocytopenia. Secondary outcomes included eltrombopag-related adverse events (especially liver enzyme elevations) and bleeding or thrombotic events during follow-up. Platelet counts were measured before each chemotherapy cycle and at week 1 after eltrombopag initiation.

### 2.6. Statistical Analysis

Descriptive statistics summarized patient and treatment characteristics. Continuous variables are presented as the mean ± standard deviation or the median (interquartile range) and categorical variables as a number (percentage). Within-patient changes in platelet counts were assessed using appropriate parametric or non-parametric tests based on distribution. A two-sided *p* < 0.05 was considered statistically significant. Analyses were performed using SPSS version 26.0 (IBM, Armonk, NY, USA) and R version 4.4.2.

## 3. Results

Thirty-one patients with chemotherapy-induced thrombocytopenia were included. The median age at eltrombopag initiation was 62 years (range 41–85), and 19 patients (61.3%) were female. Most patients had advanced disease (stage IV, 23/31; 74.2%) and had received at least two prior lines of chemotherapy. Lung (*n* = 6), gynecologic/breast (*n* = 10), and pancreatic/gastrointestinal tumors were the most common primaries; less frequent tumors are summarized in Table 1.

At eltrombopag start, the median platelet count was 33 × 10^9^/L. Patients received eltrombopag for a median of 12 weeks (range 3–72) and subsequently underwent a median of 2 chemotherapy cycles (range 0–16). Under eltrombopag support, median platelet counts rose to 71 × 10^9^/L at week 1, 79 × 10^9^/L at week 4, 107 × 10^9^/L at week 6, and 99.5 × 10^9^/L at week 12. A reduction in grade 3–4 thrombocytopenia was observed among patients who remained on eltrombopag through week 12, from 80.6% (25/31) at baseline to 25.0% (4/16) at week 12 (*p* < 0.001; Table 2). The eltrombopag dose was increased in 6 patients due to a lack of response. The dose was increased to 100 mg daily in four patients and 150 mg daily in two.

Chemotherapy continuity improved in parallel with platelet recovery. By week 1, 15/31 patients (48.4%) were able to resume or continue their planned chemotherapy; by week 3, this proportion increased to 18/31 (58.1%). After two patients were discontinued because of nonresponse, 15/29 patients (51.7%) were still receiving chemotherapy at week 4. Among those remaining on study at week 6 (*n* = 23), 15 patients (65.2%) completed the intended cycle(s). At week 12, 16 patients were still receiving eltrombopag, and 11/16 (68.8%) were able to maintain on-schedule chemotherapy. Denominators at each visit reflect only patients who remained on eltrombopag and under active follow-up at that time. The number of evaluable patients decreased from 31 at baseline to 16 at week 12, largely attributable to death from progressive malignancy, treatment discontinuation, or completion of planned chemotherapy. This attrition introduces the potential for survivor bias, as patients remaining under follow-up at later visits may have represented a more clinically favorable subgroup, and any observed improvement in platelet or chemotherapy-continuation rates at weeks 6 and 12 should be interpreted with this limitation in mind.

Eltrombopag was generally well tolerated. Three patients developed grade 1–2 elevations in liver transaminases; none required treatment discontinuation or developed bilirubin elevation. No thrombotic events and no major or life-threatening bleeding episodes were observed; two patients reported minor mucocutaneous bleeding. Because of incomplete documentation across centers, the exact number of periprocedural or rescue platelet transfusions could not be ascertained.

Overall, 23/31 patients (74.2%) achieved a platelet count ≥75 × 10^9^/L at least once during treatment (“responders”), while 8/31 (25.8%) did not. Exploratory descriptive comparisons of baseline characteristics between responders and non-responders are presented in Table 3. These comparisons are strictly hypothesis-generating; no formal statistical comparisons were made; no adjustment for multiple testing was performed, and the small subgroup sizes preclude any inference about predictive factors applicable in clinical practice. During follow-up, 14 patients died, predominantly from progression of their underlying malignancy; no death was attributed to eltrombopag or to a thrombohemorrhagic complication.

## 4. Discussion

CIT remains the one myelosuppressive toxicity without a formally approved pharmacologic therapy. As a result management in routine oncology largely depends on platelet transfusion, treatment delay, or dose reduction, all of which are imperfect [1,9,10]. Many treatment protocols interrupt or attenuate chemotherapy when platelet counts approach 75–100 × 10^9^/L, despite limited evidence supporting this threshold [1,2]. Given that even modest reductions in relative dose intensity have been shown to impair response and survival across multiple solid tumors including colorectal, breast, lung, and gynecologic cancers, CIT is not a benign inconvenience but a potential inflection point that may compromise therapeutic outcomes [3,4,6,7,8].

This therapeutic gap has prompted increasing interest in TPO-RAs as selective rescue agents for CIT. Contemporary reviews and expert guidance have positioned TPO-RAs as reasonable options in carefully chosen patients, emphasizing individualized risk–benefit assessment and attention to thrombotic risk [1,17,18]. Among these agents, romiplostim has the strongest evidence base: a randomized trial and multiple large multicenter series demonstrated platelet recovery in approximately 70–80% of patients, reduced chemotherapy delays or dose reductions, and no increase in thrombotic events [12,14,15]. Similar findings emerged from a multicenter real-world cross-sectional study from China evaluating several thrombopoietic agents, including recombinant human TPO and oral TPO-RAs, which confirmed improved platelet recovery without identifying new safety concerns [16]. Together, these studies form the foundation for recent ISTH Subcommittee guidance and subsequent expert commentary recommending TPO-RA use in selected CIT phenotypes [2,17]. Direct head-to-head comparisons between TPO-RAs in CIT are limited. In clinical practice, the choice between agents is often influenced by factors such as route of administration, drug availability, reimbursement policies, and patient preference. While romiplostim is administered as a weekly subcutaneous injection and has the strongest evidence base, oral agents such as eltrombopag may offer practical advantages in selected patients, particularly in settings where access to romiplostim is limited or where oral therapy is preferred.

The clinical profile of our cohort is broadly consistent with patient populations in whom TPO-RAs have been evaluated in prior studies. Patients were heavily pre-treated, three-quarters had stage IV disease, and chemotherapy had already been delayed a median of five weeks— clinical features in which spontaneous platelet recovery between cycles may be less likely. After initiating eltrombopag 50 mg/day 5–7 days before the intended chemotherapy cycle, median platelet counts rose from 33 × 10^9^/L to >70 × 10^9^/L at week 1 and approximately 100 × 10^9^/L by week 12, with approximately 60% of patients resuming chemotherapy. These findings are broadly consistent with those observed in randomized studies of eltrombopag in carboplatin/paclitaxel-based [20] and gemcitabine-based [19,21] regimens, where eltrombopag increased pre-chemotherapy platelet counts, reduced grade 3–4 CIT, and lowered the incidence of treatment delays—although, as in avatrombopag trials, strict composite endpoints were sometimes unmet because of spontaneous recovery in placebo arms.

The safety profile observed in our real-world series likewise aligns with prior clinical experience. Only low-grade, transient transaminase elevations occurred, no patient discontinued eltrombopag for hepatotoxicity, and no thrombotic events or major bleeding episodes were recorded. These observations are consistent with results from randomized eltrombopag trials and subsequent Asian and European series employing oral TPO-RAs with gradual titration [16,19,20,21]. Given the concomitant use of potentially hepatotoxic chemotherapy and underlying malignancy, a causal relationship between eltrombopag and transaminase elevations cannot be definitively established.

A broader question emerging from recent literature is how best to select among available TPO-RAs. The international phase 3 trial of avatrombopag achieved its biologic objective—increasing the proportion of patients reaching platelet levels compatible with chemotherapy continuation—but did not meet its composite primary endpoint, largely because the placebo arm exhibited unexpectedly high rates of spontaneous recovery between cycles [22]. Subsequent analyses and expert reviews highlighted two key insights: (i) thrombopoietic agents consistently reduce grade 3/4 CIT, improve nadir platelet counts, and decrease transfusion requirements; and (ii) effect sizes diminish when spontaneous recovery in control arms is pronounced [22,23]. Notably, eltrombopag has been reported to perform favorably in metrics related to on-schedule chemotherapy delivery, whereas avatrombopag showed a particularly favorable thromboembolism profile [22,23]. Al-Samkari’s 2025 commentary synthesizes these observations, emphasizing that TPO-RA therapy is most justifiable in patients with persistent or recurrent CIT despite chemotherapy dose modification, particularly in patients receiving highly myelosuppressive regimens such as platinum- or gemcitabine-based therapies—clinical scenarios that closely resemble the characteristics of our study population [17]. Importantly, our cohort excluded patients with alternative causes of thrombocytopenia such as bone marrow infiltration, thereby focusing on a more homogeneous population with chemotherapy-related platelet suppression.

Our study provides real-world evidence on the use of eltrombopag in a setting where dosing strategies are constrained by regulatory frameworks, necessitating initiation at 50 mg/day with subsequent titration. This provides insight into a step-up approach that is underrepresented in clinical trials and may better reflect routine practice in resource-variable settings.

Our findings should also be interpreted alongside the expanding real-world experience with thrombopoietic agents in resource-variable settings. Centers in China and Europe have reported successful use of eltrombopag, romiplostim, recombinant human TPO, and newer agents such as hetrombopag in CIT rescue, achieving meaningful platelet recovery without new safety signals [15,16,24,25]. Parallel evidence from multiple studies confirms that loss of RDI—whether driven by neutropenia, anemia, or thrombocytopenia—is associated with inferior survival [3,4,5,6,7,8]. The ability to resume on-schedule chemotherapy after a 3–5-week CIT-related interruption may be clinically relevant. This consideration is particularly important in heavily pre-treated patients with advanced disease, in whom therapeutic windows may be limited due to ongoing disease progression.

This study has the inherent limitations of a retrospective, single-arm design, including small sample size, heterogeneity of solid tumors, incomplete capture of platelet transfusions, and attrition over long-term follow-up. Incomplete documentation of platelet transfusions across participating centers limited our ability to determine whether the observed platelet recovery translated into reduced transfusion requirements. This is a meaningful gap: transfusion burden is not merely an administrative metric but a direct indicator of clinical benefit relevant to patients’ quality of life and healthcare resource use. Future studies should treat transfusion independence or reduction as a pre-specified secondary endpoint to fully characterize the clinical utility of TPO-RA therapy in CIT. In addition, important baseline clinical variables such as performance status, comorbidities, detailed radiotherapy characteristics, and comprehensive laboratory parameters were not consistently available across centers, further limiting adjustment for potential confounders and the interpretability of the findings. In addition, subgroup comparisons were exploratory and purely descriptive, and should not be interpreted as evidence of predictive associations because of the small sample size and the very limited number of patients in several subgroups. The number of evaluable patients decreased from 31 at baseline to 16 at week 12, largely due to death from progressive malignancy, treatment discontinuation, or completion of chemotherapy. This introduces the potential for attrition-related selection bias and survivor bias, as patients remaining under follow-up at later time points may have represented a more clinically favorable subgroup, potentially leading to overestimation of treatment efficacy. Bone marrow examinations were not systematically performed across the cohort. While major marrow involvement was excluded clinically and radiologically via PET/CT, we recognize that PET/CT is not scientifically equivalent to a bone marrow biopsy, as certain solid tumor infiltrations can be PET-negative and diffuse marrow uptake patterns can be nonspecific. Additionally, our data lacked detailed chemotherapy regimen analysis, formal stratification based on the specific thrombocytopenic potential of the regimens, and precise chemotherapy dose-intensity calculations.

Where does eltrombopag fit relative to other available TPO-RAs in CIT? Eltrombopag and romiplostim share a comparable biological rationale and broadly similar rates of platelet recovery, though direct comparative data in CIT are lacking. Avatrombopag achieved its biologic objective in the phase 3 trial but narrowly missed the composite primary endpoint because of unexpectedly high spontaneous platelet recovery in the placebo arm [22,23]. Expert analysis suggests that eltrombopag may be particularly advantageous for metrics related to on-schedule chemotherapy delivery, whereas avatrombopag demonstrated a notably favorable thromboembolism profile [22,23]. Hetrombopag, evaluated in a randomized phase 2 study in patients with advanced solid tumors, also achieved meaningful platelet recovery with an acceptable safety profile [24]. In practice, the choice between agents in Türkiye is constrained by the regulatory framework: eltrombopag carries approval for ITP and aplastic anemia, while romiplostim is approved solely for ITP, leading to selective off-label use of eltrombopag when a TPO-RA is clinically warranted for CIT. Our cohort illustrates a step-up dosing approach starting at 50 mg/day with titration up to 150 mg/day, a strategy that is underrepresented in clinical trials but likely reflects routine practice in many resource-limited settings.

## 5. Conclusions

In this real-world multicenter cohort, platelet counts increased modestly following the initiation of eltrombopag, allowing some of the patients to resume or maintain their scheduled chemotherapy regimens with a manageable safety profile. Given the retrospective, single-arm design and the descriptive nature of these findings, definitive conclusions regarding causal efficacy cannot be drawn. These observational data serve as hypothesis-generating insights that highlight the need for prospective controlled trials to characterize the clinical role, optimal dosing, and long-term safety of oral TPO-RAs in CIT.

## Figures and Tables

**Table 1:** Demographic, clinical, and treatment data of the patients.

Baseline Characteristics of the Patients	Number (%)
Gender	
Female	19(61.3)
Male	12(38.7)
Age (median, range)	62 (21–85)
Solid Tumor type	
Lung	6 (19.4)
Breast	5 (16.1)
Gynecologic	5 (16.1)
Pancreas	4 (12.9)
Stomach	3 (9.7)
Prostate	2 (6.5)
CNS	2 (6.5)
Head & Neck	1 (6.5)
Mesothelioma	1 (3.2)
Bladder	1 (3.2)
Testis	1 (3.2)
Stage	
II	2 (6.4)
III	6 (19.4)
IV	23 (74.2)
Number of prior Chemotherapy regimens (median, range)	2 (1–6)
Duration of Chemotherapy delay in weeks (median, range)	5 (3–18)
Platelet count before the start of Eltrombopag (median, range) × 10^9^/L	33 (7–70)
Duration of Eltrombopag use (median, range) weeks	12 (3–72)
Number of chemotherapy cycles after eltrombopag (median, range)	2.5 (0–16)
Platelet count at first week of eltrombopag (median, range) × 10^9^/L	71(10–269)
Platelet count at 4th week of eltrombopag (median, range) × 10^9^/L (*n*:19)	79 (27–287)
Platelet count at 6th week of eltrombopag (median, range) × 10^9^/L (*n*:14)	107 (17–248)
Platelet count at 12th week of eltrombopag (median, range) × 10^9^/L	99.5 (21–260)

**Table 2:** Grade of thrombocytopenia during the study period.

Grade of the Thrombocytopenia	Platelet Count Before the Eltrombopag (*n* = 31)	Platelet Count at the First Week of Eltrombopag (*n* = 31)	Platelet Count at the Third Week of the Eltrombopag (*n* = 31)	Platelet Count at the 4th Week of Eltrombopag (*n* = 29)	Platelet Count at the 6th Week of Eltrombopag (*n* = 23)	Platelet Count at the 12th Week of Eltrombopag (*n* = 16)
Grade 0	0	9	9	10	12	8
Grade 1	0	6	9	5	3	3
Grade 2	6	7	2	7	3	1
Grade 3	16	6	9	7	4	2
Grade 4	9	3	2		1	2

**Table 3:** Baseline characteristics of responders and non-responders: exploratory descriptive analysis.

	Responders (*n* = 23)	Non-Responders (*n* = 8)
Gender	Female 15 (65.2%)	Female 4 (50%)
	Male 8 (34.8%)	Male 4 (50%)
Age (median-min–max)	63 (41–85)	59.5 (21–72)
History of Radiotherapy	3 (13%)	4 (50%)
*n* = 7		
Bone Metastasis	4 (17.4%)	2 (25%)
*n* = 6		
Solid tumor type	Breast 5 (21.7%)	Lung 5 (37.5%)
	Pancreas 4 (17.4%)	Head & Neck 1 (12.5%)
	Gynecologic 4 (17.4%)	Testis 1 (12.5%)
	Stomach 4 (17.4%)	Gynecologic 1 (12.5%)
	Prostate 2 (8.7%)	
	CNS 1 (4.3%)	
	Bladder 1 (4.3%)	
	Mesothelioma 1 (4.3%)	
	Lung 1 (4.3%)	
Stage		
II	2 (8.7%)	0
III	4 (17.4%)	2 (25%)
IV	17 (72.9%)	6 (50%)

## Data Availability

The datasets generated and/or analyzed during the current study are not publicly available because of institutional regulations but are available from the corresponding author on reasonable request.
